# The Whole-Genome and Transcriptome of the Manila Clam (*Ruditapes philippinarum*)

**DOI:** 10.1093/gbe/evx096

**Published:** 2017-05-13

**Authors:** Seyoung Mun, Yun-Ji Kim, Kesavan Markkandan, Wonseok Shin, Sumin Oh, Jiyoung Woo, Jongsu Yoo, Hyesuck An, Kyudong Han

**Affiliations:** 1 Department of Nanobiomedical Science & BK21 PLUS NBM Global Research Center for Regenerative Medicine, Dankook University, Cheonan, Republic of Korea; 2 DKU-Theragen Institute for NGS Analysis (DTiNa), Cheonan, Republic of Korea; 3 TheragenETEX Bio Institute, TheragenETEX Inc., Suwon, Republic of Korea; 4 Division of Marine-Bio Research, National Marine Biodiversity Institute of Korea, Seocheon-gun, Republic of Korea

**Keywords:** *Ruditapes philippinarum*, de novo assembly, transcriptome, repeat elements

## Abstract

The manila clam, *Ruditapes philippinarum*, is an important bivalve species in worldwide aquaculture including Korea. The aquaculture production of *R. philippinarum* is under threat from diverse environmental factors including viruses, microorganisms, parasites, and water conditions with subsequently declining production. In spite of its importance as a marine resource, the reference genome of *R. philippinarum* for comprehensive genetic studies is largely unexplored. Here, we report the de novo whole-genome and transcriptome assembly of *R. philippinarum* across three different tissues (foot, gill, and adductor muscle), and provide the basic data for advanced studies in selective breeding and disease control in order to obtain successful aquaculture systems. An approximately 2.56 Gb high quality whole-genome was assembled with various library construction methods. A total of 108,034 protein coding gene models were predicted and repetitive elements including simple sequence repeats and noncoding RNAs were identified to further understanding of the genetic background of *R. philippinarum* for genomics-assisted breeding. Comparative analysis with the bivalve marine invertebrates uncover that the gene family related to complement C1q was enriched. Furthermore, we performed transcriptome analysis with three different tissues in order to support genome annotation and then identified 41,275 transcripts which were annotated. The *R. philippinarum* genome resource will markedly advance a wide range of potential genetic studies, a reference genome for comparative analysis of bivalve species and unraveling mechanisms of biological processes in molluscs. We believe that the *R. philippinarum* genome will serve as an initial platform for breeding better-quality clams using a genomic approach.

## Introduction

The seawater bivalve *Ruditapes philippinarum*, known as the manila clam or short-necked clam is a most important aquaculture species, having great commercial value and a worldwide distribution. Global production of cultured or farmed bivalve molluscs is ∼16.1 million tons per year, accounting for 22.8% of global freshwater aquaculture production in 2015 (FAO 2016). The *R. philippinarum* is largely distributed on the southern shores of the Korean Peninsula and is one of the important marine resources which determines the production rate of shellfish on the west coast in South Korea. China is the largest producer of manila clam followed by Japan and South Korea. The consumption of manila clam accounts for 0.175 kg per person per year and the consumption rate is increasing alongside its aquaculture production since 1991 in South Korea. However, exogenous influences such as infections by virus, bacteria, and perkinsus parasite and increasing water temperature have become major threats for this species especially in aquaculture production (Beaz-Hidalgo et al. 2010; Dang et al. 2009; Kim et al. 2008; Paillard et al. 2004). Remarkably, the occurrence of perkinsosis is considered as an inveterate problem in South Korea, where dramatic drop in aquaculture production has ensued (Park et al. 2006, 2010). Thus, the genome survey for *R. philippinarum* is needed for further comparative and functional studies of bivalve species and their production.

The completion of the pearl oyster genome sequence has accelerated studies on the genomes of other members of the bivalve family (Takeuchi et al. 2012, 2016; Zhang et al. 2012). To date, genomic and transcriptomic analysis of many other bivalve species focusing on the identification of candidate genes related to immunological defense (Ertl et al. 2016; Moreira et al. 2012; Nam et al. 2016; Rao et al. 2015), response to benzo (a) pyrene toxicity (Liu et al. 2014b), shell color (Yue et al. 2015), reproduction (Li et al. 2016; Patnaik et al. 2016; Teaniniuraitemoana et al. 2014), and environmental stress (Milan et al. 2011) in order to understand the biological mechanisms involved in the above factors. Recently, transcriptome analysis of *R. philippinarum* using 454 pyrosequencing technology revealed putative members of immunological defense genes involved in complement cascades, apoptosis, and the toll like signaling pathways (Moreira et al. 2012).

The lack of genomic resources coupled with poor understanding of molecular and biochemical processes have hindered advances in aquaculture productivity of marine bivalves. Understanding gene functions and their effects on phenotypes will be fundamental for selective breeding programs and disease control. Likewise, the whole-genome assembled data are essential in order to identify trait-specific loci using GWAS and for genomic selection breeding approaches. To this end, whole-genome sequencing has been conducted in several molluscs species, including *Crassostrea virginica* (Gomez-Chiarri et al. 2015), *Crassostrea gigas* (Gerdol et al. 2015), *Lottia gigantea* (Simakov et al. 2013).

In this current study, we sequenced the whole-genome and transcriptome (three tissues) of *R. philippinarum*, using the Illumina HiSeq 2500 platform. To construct the *R. philippinarum* whole-genome, we utilized short-insert paired-end (PE) sequencing, long-insert mate-pair (MP) sequencing, and TruSeq Synthetic Long-Read (TSLR) sequencing technologies (McCoy et al. 2014), as marine molluscs frequently show high level of heterozygosity in their genomes (David 1998). Consequently, to improve accuracy of gene prediction, we incorporated *R. philippinarum* transcriptome data for annotating gene sets in the assembled genome. Our study provides the basic knowledge for understanding genomic features of *R. philippinarum* and our data will be useful for further comparative, systematic, and functional genomics studies in bivalve species.

## Results and Discussion

### De Novo Assembly of *R.philippinarum*

To provide comprehensive genetic information and genomic architecture of *R. philippinarum*, we used parallel sequencing approaches to build a high-quality de novo assembly using various sizes of sequencing libraries. The DNA samples extracted from four different tissues (mouth, foot, gill, and adductor muscle) were used to construct short-insert PE, long-insert MP libraries and TSLR libraries (McCoy et al. 2014). The PE sequencing with 500 bp insert libraries generated a total of 43.03 Gb sequence data with the Illumina HiSeq 2500 platform. On the basis of a *K*-mer frequency analysis (*K* = 17), we estimated the genome size of *R. philippinarum* was ∼1.37 Gb (see supplementary fig. S1 and table S1, Supplementary Material online). Then, we exploited a combination of two MP libraries (5 and 10 kb) for better de novo assembly. For the 5 and 10 kb long-insert MP libraries, a total of 21.76 Gb and 25.15 Gb high-quality sequencing data, respectively, were produced with a quality score > Q30 (see supplementary table S2, Supplementary Material online). Sequence reads from PE and MP were assembled using SOAPdenovo v2.04 (Luo et al. 2012) into 298,671 scaffolds with an N50 length of 32.7 kb and the gaps in the scaffolds were subsequently filled with the Illumina reads by using GapCloser 1.12 (table 1; Luo et al. 2012).

**Table 1:** Statistics of De Novo Assembly for the *R. philippinarum* Genome

*Ruditapes philippinarum* Assembly	No. of Sequences	Total Bases	Longest (kb)	N50 (kb)	N90 (kb)
Short-read assembly					
Contig (PE)	4,861,413	2,257,620,557	96,344	3,333	128
Scaffold (PE + MP)	298,671	2,478,800,284	474,845	32,797	5,215
Long-read assembly					
Contig (TSLR)	247,445	2,206,994,927	149,501	12,971	4,470
Total-read assembly					
Scaffold (PE + MP + TSLR)	223,851	2,561,070,351	572,939	48,447	7,827
HaploMerger	13,411	1,078,771,101	1,050,406	119,518	39,029

Furthermore, to improve the quality and length of de novo assembled scaffolds from PE and MP sequencing data, we applied TSLR technology which is able to generate synthetic long-read data from short-read sequence. As shown in our methods, the key processes for TSLR library construction were adopted using the technique recently introduced in the study of the *Botryllus schlosseri* genome (Voskoboynik et al. 2013). Four independent TSLR libraries produced 4,303,423 reads covering 11,766,512,411 bp (11.7 Gb) of sequence data (see supplementary table S2, Supplementary Material online). A total of 894,934,711 reads produced by PE and MP sequencing were assembled into 247,445 TSLR contigs. Finally, the assembled whole-genome of 2.56 Gb for *R. philippinarum* was anchored by reconstructing TSLR data of 223,851 scaffolds with N50 length of 48.4 kb using SSPACE-LongRead v1.1 (table 1; Boetzer and Pirovano 2014). Hence, with an enormous sequencing data (101,720,272,499 bp) from three types of library construction allowed us to improve *R. philippinarum* de novo genome assembly and thus a high coverage (around 39.7× depth) of the whole-genome was obtained.

### Heterozygous Features in the *R.philippinarum* Genome

In spite of preparing high quality data, our results were not likely to satisfy N50 length for a whole-genome assembly due to the high frequency of heterozygosity in the *R. philippinarum* genome. The *K*-mer analysis of *R. philippinarum* genome exhibited two major peaks, which indicates a high rate of heterozygosity (see supplementary fig. S1, Supplementary Material online), similar to the *Ciona savignyi* genome (Small et al. 2007). Heterozygosity is a common feature in most eukaryotic organisms and generates sequencing assembly error due to single-nucleotide polymorphisms and micro-insertion or deletions between heterozygous alleles. To better understand sample sequencing quality and contamination, we performed taxonomy profiling with assembled contigs and scaffolds against NCBI taxonomy database (Altschul et al. 1990; Federhen 2015). Approximately 86% of the aligned sequences against NCBI nt database were identified in six clams belonging to marine bivalve mollusk (see supplementary fig. S2, Supplementary Material online). Forty-five percent of assigned sequences were correctly matched to given sequence data for manila clam in NCBI nt database (Taxon ID: 129788). To decrease the complexity of the *R. philippinarum* genome by heterozygosity, we made an attempt to alternatively reconstruct de novo assembly using HaploMerger (Huang et al. 2012). This approach has provided slightly better assembly results giving a scaffold N50 length of 119.5 kb (13,411 scaffolds), greater than the earlier assembly results because excessive differences between alleles were discounted as false assembly structure. We thus obtained ∼1.07 Gb size of the assembled *R. philippinarum* genome, close to the estimated genome size (1.37 Gb; table 1 and see supplementary table S1, Supplementary Material online). This result supports fairly high heterozygosity rate in the *R. philippinarum* genome. However, here, we adopted the assembly data using SSPACE-LongRead v1.1 for further comprehensive genetic analyses of *R. philippinarum* genome as we wished to avoid spurious merging of contigs in the genome by Haplomerger.

### Repeat Composition and Simple Sequence Repeat (SSR) Discovery

To investigate repeat composition in the *R. philippinarum* genome, we examined repeat components through homology and de novo-based approaches. First of all, we identified a total of 26.38% of repeats (e.g., transposable elements) in the genome using the RepeatMasker program (Smit and Green 1996–2010). However, 17.09% of numerous regions in the genome were sorted as unclassified or unknown repeats. Around 5.09% and 3.44% of the genome are classified into DNA transposons and retrotransposons, respectively. Comparatively, rolling-circle (RC) transposons belonging to DNA transposons are the most abundant repeats (3.01%) in the *R. philippinarum* genome (see supplementary table S3, Supplementary Material online). We further investigated the features of SSRs in order to provide the basis data for polymorphic information of clam and also to suggest molecular marker candidates. On the basis of the screening results, dinucleotide repeats showed high frequency as 156,972 copies and accounted for 61.29 percentage per million bases. In contrast, the number of each hepta to deca-nucleotide repeat is shown around <100 copies (see supplementary table S4, Supplementary Material online). Among dinucleotide repeats, the highest frequency (109,191 copies) was AT (AT/TA) motif and the lowest frequency (1,102 copies) was CG (CG/GC) motif. In trinucleotide repeats, we were able to observe high occupancy of the (A + T)-rich motif including the combinations with AAT, AAG, AAC, ATG, and AGT (see supplementary table S5, Supplementary Material online). The (A + T)-rich motifs are around 20 times higher than the (C + G)-rich motifs. The AAT (AAT/ATA/TAA/ATT/TTA/TAT) motifs are highly distributed among the genome which accounting for 44.27% among trinucleotide repeats. On the basis of these data, we identified a total of 7,368 primers for SSR targets, which can be used for further polymorphism screening across the congener species of *R. philippinarum* (see supplementary table S6, Supplementary Material online).

### Gene Prediction and Annotation

Leveraging recent advances in NGS technologies, a huge amount of sequencing data is recently accumulated and utilized for extensive researches in evolutionary genetics (Foote et al. 2015). However, in comparison with mammalian genome studies, understanding marine mollusk genome architecture is still in its infancy. Here, we have tried to provide a commendable resource for study of marine bivalve genomics. With repeat-masked genome sequencing data, we accomplished gene prediction and structural-annotation by homology based search prior to determination of gene set with transcriptome data. At first, we sought to comprehensively describe the noncoding RNAs (ncRNA) with the purpose of building better coding gene models. By homology-based Blast search, a total of 240 rRNA copies were matched with 29,746 bp. In addition, 5,360 tRNA copies accounting for 0.02% of its genome were estimated using tRNAscan-SE tool (Lowe and Eddy 1997). The miRNAs with 20,118 copies (2,071,902 bp) and snRNAs with 1,756 copies, respectively were found using INFERNAL (see supplementary table S7, Supplementary Material online; Nawrocki et al. 2015; Nawrocki and Eddy 2013).

To conduct empirical analysis for gene identification and prediction, we used three different gene prediction methods (ab initio, homology-based, and RNA-seq-based annotation) and the results from each method were used for the construction of a final gene set using the Hidden Markov Model (HMM)-based gene prediction program, AUGUSTUS (Stanke et al. 2004). As a result, a total of 108,034 gene models including 1,932 isoforms were predicted as described in the methods section. Among them, 98,442 gene models were supported by our transcriptome data. We further analyzed the entire predicted genes comprised around 21.58% of the genome (552.9 Mb) with 451,049 predicted exons (4.09%) of the genome. Average length of genes was calculated as 5,117 bp. The detail of summary statistics for gene prediction is reported in table 2.

**Table 2:** Results of Gene Prediction for the *R. philippinarum* Genome

Classification	Quantification
Total no. of gene models predicted	108,034
Unique gene models (no.)	106,102
Genes with isoforms (no.)	1,932
RNA-Seq supported gene model (no.)	98,442
Average gene length (bp)	5,117 bp
Total bases of gene models (Mb)	552.90 Mb
Genes in the draft genome (%)	21.58%
No. of exon	451,049
Average no. of exon per gene	4.17
Average exon length (bp)	232 bp
Exons in the draft genome (%)	4.09%
No. of intron	343,015
Average no. of intron per gene	3.17
Average intron length (bp)	1,230 bp
Introns in the draft genome (%)	16.48%

Gene annotation using Uniprot, NCBI nonredundant (NCBI nr), and InterProScan database were used as sources for protein sequences and also to find its biological functions (Pruitt et al. 2007; Quevillon et al. 2005; UniProt 2015). Among the 108,034 gene models 45,595, 70,136, and 70,061 genes were predicted by the Uniprot, the NCBI nr, and the InterProScan database, respectively. Consequently, 80,449 gene models (74.47%) were annotated with respective gene identifiers and 44,569 gene models (41.25%) were common on all three identifiers (see supplementary fig. S3, Supplementary Material online). Here, we first reported the consensus gene sets of *R. philippinarum* which are predicted with highly accurate de novo genome assembly data. Over 91% of the predicted gene models were verified by comparing with transcriptome data obtained from three different tissues. These results could be useful for the ecological and immunological studies of infectious disease in marine environments and also provide a better understanding of the evolutionary relationship between bivalve molluscs.

### Comparative Analysis of Orthologous Gene Families

To investigate the detailed gene annotation of *R. philippinarum*, we performed orthologous gene clustering analysis of all predicted *R. philippinarum* genes with oyster, snail, octopus, drosophila, lancelet, mouse, and human. Notably, we found that *R. philippinarum* genome contains 4,605 orthologous gene families. Among them, 3,033 orthologous gene families are shared with all seven genome and 154 gene families were annotated only in the *R. philippinarum* genome (see supplementary table S8, Supplementary Material online). In addition, we identified highly enriched gene families in the *R. philippinarum* genome by comparing with the other genomes. We found a total of 1,042 gene families that represent high copy number of genes in the *R. philippinarum* genome. To determine the significant expansion of gene families in the *R. philippinarum*, we selected gene families using defined criteria as described in methods section. Thus, we identified that a total of 62 gene families corresponding to 5,163 gene models contain at least three times higher copy number in the *R. philippinarum* than other genomes (see supplementary table S9, Supplementary Material online).

**Fig. 1.—:**
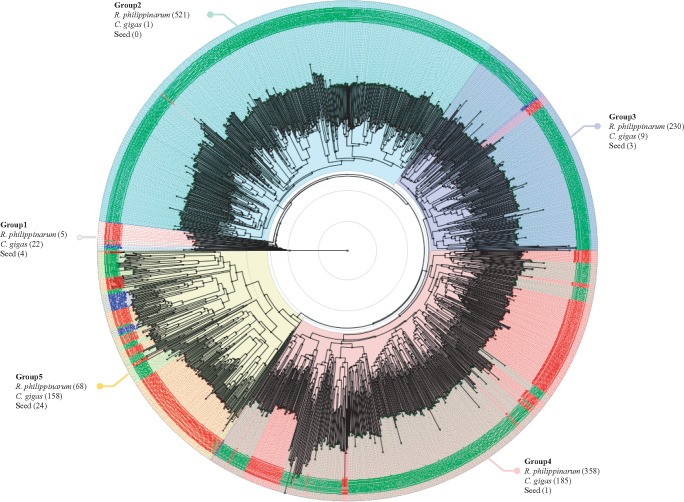
Phylogenetic tree of C1qDC genes. The tree was reconstructed by BLOSUM62 and the Neighbor-Joining methods using the Jalview v2.10.1 program and visualized by using Figtree tool v1.4.3. Each taxon of C1qDC genes in *R. philippinarum*, *C. gigas*, and PFam seeds are written in green, red, and blue letter, respectively. A total of 1,589 C1qDC genes were used for this analysis and divided into five groups. The number in brackets indicates the number of C1qDC genes in *R. philippinarum*, *C. gigas*, and PFam seeds, clustering to each group.

Among 62 gene families, the gene family that encodes complement C1q gene (PF00386) was significantly enriched in *R. philippinarum*. The expansion of gene family can be due to duplication of a single gene and we assumed that the C1q domain gene family has been remarkably expanded in *R. philippinarum* similar to *C. gigas* (Gerdol et al. 2015; Takeuchi et al. 2016). On the other hand, a lower number of C1qDC genes were found in snail, octopus, drosophila, lancelet, mouse, and human genomes. This gene family member is 12 and 3 times expanded more than human and oyster, respectively. This C1q domain containing (C1qDC) proteins are known as an important component in the innate immune response and play crucial roles for pathogen recognition and activation of complement system (Kishore and Reid 2000; Liu et al. 2014a; Tom Tang et al. 2005). The C1qDC proteins consist of various pattern recognition domains to bind various self and nonself-ligands, including retroviral envelope proteins, peptidoglycan, β-amyloid fibrils, phospholipids, and apoptotic cells (Bohlson et al. 2007; Wang et al. 2012). To investigate the C1q domain copies in *R. philippinarum* and *C. gigas* genomes, we performed multiple sequence alignment with C1q Pfam seed alignments (PF00386; Finn et al. 2016). A total of 1,589 C1qDC genes including C1q seed sequences were implicated in amino acid sequence alignment analysis (see supplementary file S1, Supplementary Material online). In case of phylogenetic analysis, the predicted C1qDC genes in *R. philippinarum* were clustered together with C1qDC genes from *C. gigas*, but seem to be highly diverged from other known C1q genes. The C1qDC genes were substantially divided into five different groups based on Pfam seed clustering (fig. 1). For the “group 5”, 24 C1q seeds from human (seven seeds), mouse (9), cattle (1), swine (1), horse (1), rabbit (1), chicken (1), frog (1), and zebrafish (2) were clustered with 68 and 158 C1qDC genes from *R. philippinarum* and *C. gigas*, respectively. Whereas, the majority of C1qDC genes in the both bivalve genomes is scattered across in other groups. Especially in group 4, a high number of C1qDC genes in *R. philippinarum* and *C. gigas* are clustered, but branched off with many known vertebrate C1qDC genes, suggesting that these C1qDC genes in the both bivalve genomes have undergone independent expansion in their lineage. Unlike groups 4 and 5, groups 2 and 3 are likely that a lineage-specific expansion of C1q genes has occurred in the *R. philippinarum* lineage. These results suggested that both bivalves have a high copy number of C1qDC genes, but these genes have evolved separately by gene duplication events specific to each lineage. In support of the aforesaid studies and several recent studies on the C1q gene have also hypothesized an expansion of the C1q domain gene family in *R. philippinarum* which may account for its immunological defense mechanisms against various biotic and abiotic factors in the marine habitats (Gerdol et al. 2011, 2015; Philipp et al. 2012).

We also further investigated the relevant gene ontology (GO) categories of these expanded 62 gene families based on predicted PFam entries (Mitchell et al. 2015). Of the 62 gene families, 22 gene families are matching with at least one GO term. Considering cellular components, three gene families such as opioid growth factor receptor (OGFr, GO:0016020, 29 genes), interferon-inducible GTPase (IIGP, GO:0016020, 145 genes), and interleukin-17 (IL-17, GO:0005576, 43 genes) were only identified as components of membrane and extracellular region. Twenty-two gene families were corresponding to 17 GO terms of molecular function and the most represented group was related with DNA binding activity (GO:0003677) such as helix-turn-helix (HTH), RNA polymerase II transcriptional coactivator p15 also known as positive cofactor 4 (PC4), transposase, and phage integrase family. Within the biological process classification, 15 out of 62 gene families were correlated with 14 GO categories (see supplementary fig. S4, Supplementary Material online).

**Fig. 2.—:**
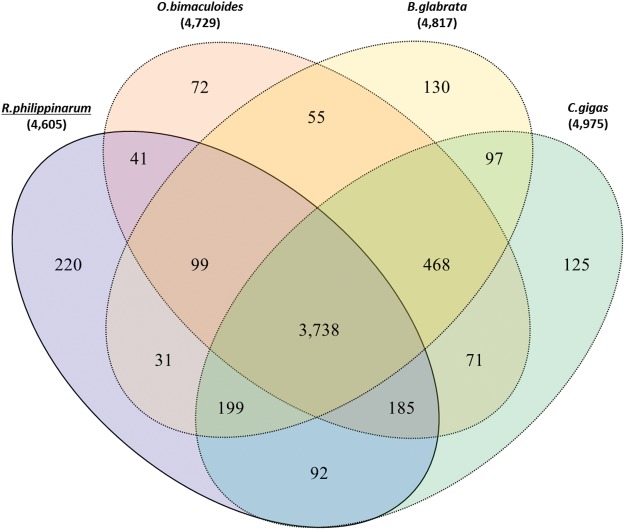
Orthologous gene clusters in the Mollusca lineage. Venn diagram shows the number of unique and shared gene families among the four Mollusca genomes (manila clam, octopus, snail, and oyster).

**Fig. 3.—:**
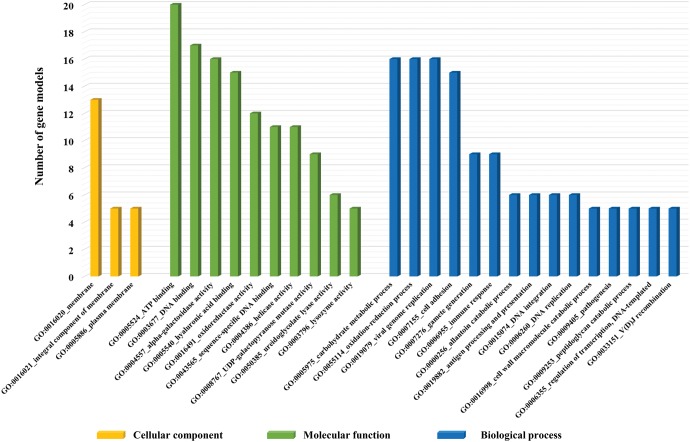
Gene ontology (GO) categories of unique gene families in *R. philippinarum* among four Mollusca. The functions of 68 gene families were classified and subdivided into a total of 93 GO terms. However, the majority of gene families is only represented in this figure as follows three main categories: Cellular components (yellow), molecular function (green), and biological process (blue). The *x* axis denoted the related functional categories and the *y* axis denoted the number of gene models, which are associated with each GO categories.

Alternatively, we compared clustered gene families among the four genomes (i.e., clam, oyster, snail, and octopus) belonging to phylum Mollusca. The results revealed that 3,738 out of 4,605 orthologous gene families (81.1%) are shared and 92 gene families (1.9%) are exclusively shared only by two bivalve genomes (Clam and Oyster). An inspection of orthologous gene family within molluscs revealed that 220 gene families are more specific to the *R. philippinarum* genome (fig. 2 and see supplementary table S10, Supplementary Material online). Deciphering the function of gene families enriched only in the *R. philippinarum* genome, we inspected its GO terms using aforementioned method. A total of 30, 56, and 171 gene families were considered as functional gene families (14 cellular components, 41 molecular functions, and 38 biological process categories, respectively). The majority of the cellular components was related to membrane (GO:0016020, 30%). The DNA binding (GO:0003677) and ATP binding (GO:0005524) molecular processes represent 17% and 9.7% of the total molecular functions, respectively. For the biological process category, immune response (GO:0006955) and oxidation–reduction (GO:0055114) process-related gene families were highly enriched in the *R. philippinarum* genome (fig. 3). Remarkably, oxidation–reduction process is an important metabolic pathway for bivalves under hypoxia condition where they seal their valves when exposed to low tide or oxygen concentration. Besides, all living organisms in hypoxic condition require anaerobic energy production through activity of reductase enzymes (Almeida and Mascio 2005; de Zwaan and Wijsman 1976; Schick et al. 1986). In addition, the reductase enzymatic activity is very effective defense system to reduce damage on cellular components (nucleic acids, lipids, proteins, and polysaccharides) by reactive oxygen species (ROS; Manduzio et al. 2005; Livingstone 2001). Through comparison analysis between the four mollusc genomes, we described five reductase gene families were only observed in *R. philippinarum* as follows: Pyridine nucleotide-disulphide oxidoreductase (PNDR), hydroxylamine reductase/Ni-containing CO dehydrogenase, violaxanthin de-epoxidase (VDE), polyphenol oxidase (PPO), and Rieske-type gene families. Accordingly, we propose that these oxidation–reduction-related gene families in *R. philippinarum* can contribute in antioxidant systems against oxidative stress, caused by increasing of the ROS. Through comparative analysis along with orthologous gene families, we suggest that the several expanded gene families in *R. philippinarum* may contribute to environmental adaptations and provided genetic differences between them.

### Whole-Transcriptome Analysis

**Fig. 4.—:**
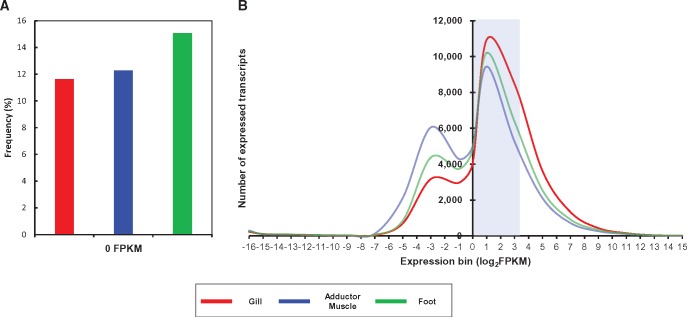
Bioinformatic analysis for transcript expression profile. (*A*) Frequency of transcripts with 0 FPKM in each tissue. (*B*) Distribution of FPKM values for transcripts expressed in each tissue. The *x* axis and *y* axis indicate the number of expressed transcripts and log-transformed FPKM values of transcripts (FPKM > 0) from three tissues, respectively. The blue box denotes the majority of transcripts (1 < FPKM < 10).

In support of genome annotation and also to understand genetic features of *R. philippinarum*, we conducted whole-transcriptome analysis using an Illumina HiSeq 2500. A total of 216,804,402 PE reads were sequenced with an average of ∼7.2 Gb raw data from gill, adductor muscle, and foot tissues. Through filtering of low-quality reads, 201,706,234 reads with average 6.7 Gb in three tissues were finally selected and used for de novo transcriptome assembly. All the cleaned reads were assembled by Trinity assembly program with an optimized 25 bp in k-mer length (Grabherr et al. 2011). Then, the assembled data passed through supplementary step in which overlapped sequences was discarded because Trinity generates various transcripts including isoforms and chimeric transcripts. Consequently, 199,345 nonredundant unigenes (FPKM: Fragments per kilobase per million mapped fragments > 0) were constructed with mean length of unigenes was 882 bp with N50 of 1,600 bp. Among them, 112,061, 64,844, and 73,058 unigenes were highly expressed (FPKM ≥ 1) in gill, adductor muscle, and foot, respectively. To establish more accurate gene annotation, we analyzed transcriptome data along with whole-genome sequencing data. As a result, a total of 66,879 assembled transcripts with complete protein coding sequences were predicted using TransDecoder (Haas et al. 2013; table 3). Through homology searching by the BLASTx program from GenBank nonredundant (NR) and InterProScan database, we then annotated that, a total of 41,075 transcripts expressed in either of gill, adductor muscle, or foot. Transcript expression in each tissue was calculated based on the FPKM values. Among 41,075 annotated transcripts, 36,302 (88.4%), 36,026 (88.1%), and 34,886 (84.9%) transcripts are transcriptionally active (FPKM > 0) in gill, adductor muscle, and foot, respectively (see supplementary tables S11 and S12, Supplementary Material online). The frequency of unexpressed transcripts in foot tissue was marginally higher than other tissues (fig. 4). The gene expression distribution in three tissue averages 27.9% of annotated transcripts (20.1% in gill tissue to 36.3% in adductor muscle), have shown below FPKM < 1, whereas majority of transcripts was moderately expressed (1 < FPKM < 10). The gill tissue showed the dense distribution of transcripts expression, while expression distribution in adductor muscle was comparatively lower (fig. 4). To investigate the pattern of transcript expression between three tissues, we compared the top 1% ranked transcripts among the annotated transcripts, except for ribosomal protein subunits, in either tissues. A total of 263 transcripts were clustered (see supplementary fig. S5, Supplementary Material online). According to the hierarchical cluster analysis for each tissue, it is likely that adductor muscle and foot tissue have correlation with gene expression rather than gill tissue. Proteasomal ubiquitin receptor (ADRM1), which play a central role in cellular proteostasis by ubiquitin-mediated protein degradation pathway, showed highest expression in all three tissues (Jorgensen et al. 2006). In addition, elongation factor 2, translation initiation factor 5A-1, and ATP synthase subunit alpha/beta were also highly expressed in three tissues. Here, we present de novo genome annotation of *R. philippinarum*, compensated with whole-transcriptome data, and provided sufficient information for a large set of transcripts, expressed in three tissues.

**Table 3:** Summary of Whole Transcriptome Analysis and Unigene Construction

Tissue	Total RNA Reads	Filtered RNA Reads (%)	GC Rate (%)	Assembly Result				Expressed Transcript (FPKM > 0)	Expressed Transcript (FPKM > 1)	Predicted Transcripts
				Nonredundant Unigenes	Average Unigenes Length	Unigene N50 (bp)	Gene			
Gill	67,441,250	62,923,824 (93.3)	37.63	199,345	882	1,600	132,766	144,535	112,061	66,879
Adductor muscle	81,677,808	75,874,878 (92.9)	38.92					111,441	64,844	
Foot	67,685,344	62,907,532 (92.9)	37.49					108,559	73,058	

## Materials and Methods

### *Ruditapes philippinarum* Sampling and Nucleic Acid Sample Preparation

We collected healthy *R. philippinarum* samples in July, 2015 from a tidal flat farm in western sea of South Korea (36°53′21.3″N 126°22′00.6″E). We collected ∼1.5-year-old *R. philippinarum* samples by measuring morphometric characteristics such as shell length (32–33.5 mm) and weight (7–8 g). The collected *R. philippinarum* samples were dissected and frozen immediately.

The nucleic acids were extracted from two *R. philippinarum* adult individuals for sequencing library preparation of whole-genome and transcriptome, respectively. The dissected tissues were frozen in liquid nitrogen and homogenized. DNA was extracted from four tissues including mouth, gill, adductor muscle, and foot using Qiagen DNeasy Blood & Tissue Kit (Qiagen, Germany) according to the manufacture’s protocol. Extracted DNA was measured by Quant-iT BR assay kit (Invitrogen). Total RNAs were independently extracted from gill, adductor muscle, and foot using Trizol reagent (Invitrogen, USA). In order to increase purity and prevent genomic DNA contamination, we carried out RNA purification using RNeasy Mini Kit (Qiagen). Then, the quality of RNAs was checked by 28S/18S ratio, and RNA integrity number (RIN) value using TECAN Infinite F200 and Agilent Bioanalyzer 2100 system. All RNAs extracted from each three tissue shows 6.7–6.9 RIN values.

### Whole-Genome Sequencing Library Construction

PE, MP libraries were prepared following the manufacturer’s instructions. For short-read sequencing libraries, high-molecular weight genomic DNA was sheared to ∼500 bp for PE, whereas 5 kb, and 10 kb for MP libraries using a Covaris S2 Ultrasonicator system. All DNA sequencing libraries were constructed according to Illumina’s guideline. To confirm library construction, we ascertained the right size of libraries by using a 2200 TapeStation (Agilent Technologies, CA, USA). Normalization of libraries was diluted with hybridization buffer then the clusters of each library were generated on a cBot System (Illumina) by using HiSeq Rapid Duo cBot Sample Loading Kit (Illumina). Final library products were sequenced on the Illumina HiSeq 2500 using the HiSeq Rapid Paired-End Cluster Kit v2 and SBS Kit v2 (Illumina) for 100 PE sequencing.

### Synthetic Long-Read DNA Library

The synthetic long-read DNA library preparation was constructed according to the general protocol of TSLR DNA Library Prep Kit (Illumina). We digested genomic DNA into 8–10 kb fragments in g-Tube (Covaris). These fragments then went through end-repair and ligation of amplification adapters, before being diluted onto 384-well plates so that each well contains DNA representing ∼1–2% of the genome (∼200 molecules, in the case of *R. philippinarum*). Long-range PCR was used to amplify molecules within wells, followed by highly-parallel Nextera-based fragmentation and barcoding of individual well. Then, DNAs from every well were pooled from 384-well plate into a single sample, cleaned, and concentrated in order. The pooled long-read DNA libraries were conducted to check the right size pattern on a 2200 Tape Station (Agilent Technologies, CA, USA). To initiate whole-genome sequencing, the diluted libraries were under cluster generation on the cBot system (Illumina). A fully loaded flow cell was sequenced on a HiSeq 2500 (Illumina) using the HiSeq SBS and Cluster Kit v4 for 200 cycles.

### Raw Sequencing Read Filtering for De Novo Assembly

In order to secure high quality sequence reads of all sequencing libraries, we filtered out of the raw read according to criteria below.

1) Reads with ambiguous bases (represented by the letter N) or poly-A structure contents.

2) Reads with ≥40% low-quality bases (base quality ≤7) from 500 bp insert size library and reads with ≥60% low-quality bases from large insert size libraries.

3) Reads with adapter contamination: Reads with ≥10 bp aligned to the adapter sequence were filtered out (no >3 bp mismatch allowed).

4) Small insert size reads in which read1 and read2 overlapped by ≥10 bp (only 10% mismatch allowed).

5) PCR duplications (reads were considered duplicates when read1 and read2 of the two PE reads were identical).

### De Novo Assembly of the *R.philippinarum* Genome

Prior to *R. philippinarum* genome assembly, we estimated its genome size based on K-mer analysis with K-mer size of 17-bp using SOAPec v2.01 Tool. The genome size was calculated using the following formula (Genome Size = Total number of K-mer/K-mer depth). The size of *R. philippinarum* genome was estimated at around 1.37 Gb (see supplementary fig. S1 and table S1, Supplementary Material online).

With only qualified reads from PE and MP libraries, the *R. philippinarum* genome was assembled with contig construction followed by scaffolding, and gap closure. In the contig construction, the short insert library (500 bp) data were assessed to construct a *de Brujin graph* using SOAPdenovo software v2.04 with default parameters (Luo et al. 2012). Then, we discarded all erroneous data such as the clip tips, bubbles, and connection with low coverage. All qualified reads were realigned with the contig sequences. Then, we calculated the PE relationship between each pair of contigs and built the scaffolds step by step, from short insert-size to long distance PEs. The synthetic long read data were also used to promote long-range scaffolding of de novo assembly. Sequence reads generated from the synthetic long-read libraries were assessed to construct the TSLR contigs using TruSeq Long-Read Assembly tool v1.1 with default parameters (McCoy et al. 2014). Consequently, the reconstruction of scaffolds with the underlying scaffolds and TSLR contigs was conducted with SSPACE-LongRead v1.1 with default parameters (Boetzer and Pirovano 2014). Finally, the gaps between the scaffolds, mainly derived from repeats, were covered with the high quality PE information by using GapCloser tool v1.12 with default options (Luo et al. 2012). The significant heterozygosity in the *R. philippinarum* genome was likely to present assembly errors. In addition, we tested alternative assembly methods with default parameters, HaploMerger2 v 20151124 software, to counteract those issues of high level of heterozygosity in the *R. philippinarum* genome (Huang et al. 2012).

### Taxonomy Profiling

All assembled genome data were subjected to perform nucleotides sequence alignments with NCBI nr nucleotide database (Blast v2.2.29) by using megablast algorithm with an *E*-value cutoff of 1E–5 (Altschul et al. 1990). Next, taxonomy assignments by using aligned contigs and scaffolds were performed with the empirical taxonomy information from NCBI database (Federhen 2015). The visualization of taxonomy profiling were conducted with the Krona tool v2.5 (Ondov et al. 2011).

### Repeats Identification

To identify precise repeats in *R. philippinarum* genome, we applied two repeat-masking approaches including homology-based and de novo-based. The homology-based repeat search was processed with RepeatMasker software (v4.0.6) using Repbase libraries representing known repeats (Smit and Green 1996–2010). De novo repeat identification and modeling were finished by repeat finding program, RepeatModeler v1.0.8 (Price et al. 2005). SSRs were identified by using perl script on simple sequence repeat identification tool (SSRIT) (ftp://ftp.gramene.org/pub/gramene/archives/software/scripts/ssr.pl) in the *R. philippinarum* genome. SSR target primer pairs were designed with flanking sequences of SSR by using Primer 3 program with the following criteria (GC contents > 50%; product size range in 150–200 bp; annealing temperature range in 55–62 °C; primer length 18–26 bp in size; Untergasser et al. 2012).

### Prediction of ncRNAs

From de novo assembled *R. philippinarum* genome, four types of ncRNA, microRNAs (miRNAs), transfer RNAs (tRNAs), ribosomal RNAs (rRNAs), and small nuclear RNAs (snRNAs), were detected by searching databases. tRNAscan-SE with default setting was used to search for reliable tRNA positions (Lowe and Eddy 1997). To detect snRNAs and miRNAs, INFERNAL v1.1.1 was implicated in searching for putative sequences with the Rfam database (release 9.1; Nawrocki et al. 2015; Nawrocki and Eddy 2013). For rRNA predictions in the *R. philippinarum* genome, BLAST was performed which is a homology search method (Altschul et al. 1990).

### Genome Annotation

*Ruditapes philippinarum* genome annotation was performed using a combination of evidence-based gene prediction (RNA-seq and proteins) and ab initio gene prediction. First, transcript alignment was performed on the repeat masked genome data based on Tophat2 with default options (Kim et al. 2013). The aligned results with transcript data were implicated in prior gene prediction by using GeneMark-ET tool (v4.29; Hoff et al. 2016). Next, homologous proteins of other species were mapped to the genome using TBlastN (Blast v2.2.29) with an *E*-value cutoff of 1E–5 and the aligned protein sequences were implicated in prediction of the gene region using Exonerate tool v2.2.0 with default parameters (Altschul et al. 1990; Slater and Birney 2005). A final gene set of *R. philippinarum* was decided by using AUGUTUS tool v3.2.1 with default setting (Stanke et al. 2004). Gene functions were assigned according to the best alignment attained using BLASTP to the UniProt database (*E*-value cutoff of 1E–5) and INTERPRO v5.17 scan.

### Phylogenetic Analysis for C1qDC Genes

We collected C1qDC genes from the *R. philippinarum* and *C. gigas* genomes, and used the Pfam seed alignment sequences of C1q gene family (PF00386) as their conserved sequences (Finn et al. 2016). Next, the ClustalW2 multiple alignment program was used to generate the multiple amino acid sequence alignment of the C1q domain gene using default option (Larkin et al. 2007). Through the Jalview v2.10.1 program, the conserved domains of the C1q gene were selected based on the Pfam seed alignment sequences and the phylogenetic tree was constructed with the BLOSUM62 and the Neighbor-Joining methods (Waterhouse et al. 2009). The generated tree was finally visualized by using FigTree tool v1.4.3 (http://tree.bio.ed.ac.uk/software/figtree/).

### Transcriptome Sequencing

In order to construct cDNA library for RNA sequencing, mRNA was enriched by oligo-dT attached magnetic beads from a total 2 µg of RNA. Then, the purified mRNAs were sheared into short fragments and synthesized to double-stranded cDNA by reverse-transcription, immediately. Synthesized cDNA was subjected to end-repair, poly-A addition, and connected with 5′ and 3′ adaptors on both ends using the TruSeq RNA sample prep Kit (Illumina, CA). Modified mRNA fragments were separated on bluepippin 2% agarose gel cassette and the suitable fragments were automatically purified and used as templates for PCR amplification. The final products were 400–500 bp in length and were evaluated by Agilent High Sensitivity DNA kit (Agilent Technologies, USA) on the Agilent Bioanalyzer 2100 system. Subsequently, constructed libraries were sequenced using an Illumina HiSeq 2500 sequencer (Illumina, USA). All processes were conducted by TheragenETEX Bio Institute (Suwon, South Korea).

### Analysis of Transcriptome Data

Prior to mapping and assembly, the raw reads were evaluated by sequence quality score, GC content, and duplicate sequences. Raw reads filtered through in-house scripts by these criteria: First, reads including >10% of skipped bases, second, reads including >40% of bases whose quality score is <20%, third, their average quality score is <20. In addition, sequences under quality score 20 in both ends of filtered reads were also discarded due to increasing assembly quality (Martin and Wang 2011). On the basis of high-quality reads, de novo transcriptome assembly was performed using the short-read assembly software Trinity (Grabherr et al. 2011) and assembled contigs were processed with clustering using TGICL (Pertea et al. 2003) and CAP3 (Huang and Madan 1999) for unigene construction. To understand characterization and function of unigenes, coding sequence (CDS) was predicted by TransDecoder (Haas et al. 2013) and DNA and protein sequence homology searching was conducted by NCBI BlastX (2.2.28+) and InterProScan v5 (Quevillon et al. 2005).

To quantify the gene expression across three tissues, we measured the expression of unigenes with fragments per kilobase of exon per million fragments (FPKM) and calculated expression levels by CUFFLINKS with default parameters (Li and Dewey 2011). We inspected the FPKM distribution of all transcripts across samples and selected the highly expressed transcripts in each tissue. Further, clustering analysis was performed using the FPKM values followed by the heatmap was constructed using pheatmap R package (v1.0.8, available at http://cran.r-project.org/web/packages/pheatmap/index.html) with the popular clustering distance (*euclidean*) and hierarchical clustering method (*complete*) functions.

## Supplementary Material

Supplementary data are available at *Genome Biology and Evolution* online.

## Authors’ Contributions

SM, YJK, JY, HA, and KH designed the study, performed sample collection, and drafted the manuscript. SO, WS, and JW processed samples. SM, YJK, and KM analyzed and interpreted the data. All authors read and approved the final manuscript.

## Acknowledgements

This study was supported by grants from the National Marine Biodiversity Institute of Korea (2015M00500). We also thank the associate editor and the two anonymous reviewers for their critical evaluation and valuable suggestions for our manuscript.

## Supplementary Material

Supplementary Data

## References

[evx096-B1] Almeida EA, Bainy ACD, Dafre AL, Gomes OF, Medeiros MHG, Di, Mascio P 2005 Oxidative stress in digestive gland and gill of the brown mussel (Perna perna) exposed to air and re-submersed. J Exp Mar Biol Ecol. 318:21–30.

[evx096-B2] Altschul SF, Gish W, Miller W, Myers EW, Lipman DJ. 1990 Basic local alignment search tool. J Mol Biol. 215:403–410. 2231712 10.1016/S0022-2836(05)80360-2

[evx096-B3] Beaz-Hidalgo R, Balboa S, Romalde JL, Figueras MJ. 2010 Diversity and pathogenicity of Vibrio species in cultured bivalve molluscs. Environ Microbiol Rep. 2:34–43. 23765996 10.1111/j.1758-2229.2010.00135.x

[evx096-B4] Boetzer M, Pirovano W. 2014 SSPACE-LongRead: scaffolding bacterial draft genomes using long read sequence information. BMC Bioinformatics. 15:211. 24950923 10.1186/1471-2105-15-211 PMC4076250

[evx096-B5] Bohlson SS, Fraser DA, Tenner AJ. 2007 Complement proteins C1q and MBL are pattern recognition molecules that signal immediate and long-term protective immune functions. Mol Immunol. 44:33–43. 16908067 10.1016/j.molimm.2006.06.021

[evx096-B6] Dang C, 2009 Virus-like particles associated with brown muscle disease in Manila clam, *Ruditapes philippinarum*, in Arcachon Bay (France). J Fish Dis. 32:577–584. 19476559 10.1111/j.1365-2761.2009.01019.x

[evx096-B7] David P. 1998 Heterozygosity-fitness correlations: new perspectives on old problems. Heredity (Edinburgh). 80(Pt 5):531–537. 9650277 10.1046/j.1365-2540.1998.00393.x

[evx096-B8] de Zwaan A, Wijsman TC. 1976 Anaerobic metabolism in Bivalvia (Mollusca). Characteristics of anaerobic metabolism. Comp Biochem Physiol. B 54:313–324. 6196 10.1016/0305-0491(76)90247-9

[evx096-B9] Ertl NG, O’Connor WA, Papanicolaou A, Wiegand AN, Elizur A. 2016 Transcriptome analysis of the Sydney rock oyster, *Saccostrea glomerata*: insights into molluscan immunity. PLoS ONE. 11:e0156649. 27258386 10.1371/journal.pone.0156649 PMC4892480

[evx096-B10] FAO 2016 The State of World Fisheries and Aquaculture 2016. Contributing to food security and nutrition for all. Food and Agriculture Organization of the United Nations: 200 http://www.fao.org/3/a-i5555e.pdf.

[evx096-B11] Federhen S. 2015 Type material in the NCBI Taxonomy Database. Nucleic Acids Res. 43:D1086–D1098. 25398905 10.1093/nar/gku1127 PMC4383940

[evx096-B12] Finn RD, 2016 The Pfam protein families database: towards a more sustainable future. Nucleic Acids Res. 44:D279–D285. 26673716 10.1093/nar/gkv1344 PMC4702930

[evx096-B13] Foote AD, 2015 Convergent evolution of the genomes of marine mammals. Nat Genet. 47:272–275. 25621460 10.1038/ng.3198 PMC4644735

[evx096-B14] Gerdol M, 2011 The C1q domain containing proteins of the Mediterranean mussel *Mytilus galloprovincialis*: a widespread and diverse family of immune-related molecules. Dev Comp Immunol. 35:635–643. 21295069 10.1016/j.dci.2011.01.018

[evx096-B15] Gerdol M, Venier P, Pallavicini A. 2015 The genome of the Pacific oyster *Crassostrea gigas* brings new insights on the massive expansion of the C1q gene family in Bivalvia. Dev Compar Immunol. 49:59–71. 10.1016/j.dci.2014.11.007 25445912

[evx096-B16] Gomez-Chiarri M, Warren WC, Guo X, Proestou D. 2015 Developing tools for the study of molluscan immunity: the sequencing of the genome of the eastern oyster, *Crassostrea virginica*. Fish Shellfish Immunol. 46:2–4. 25982405 10.1016/j.fsi.2015.05.004

[evx096-B17] Grabherr MG, 2011 Full-length transcriptome assembly from RNA-Seq data without a reference genome. Nat Biotechnol. 29:644–652. 21572440 10.1038/nbt.1883 PMC3571712

[evx096-B18] Manduzio H, Durand F, Galap C, Leboulenger F,, Rocher B. 2005 The point about oxidative stress in molluscs. Invertebr Surv J. 2:91–104.

[evx096-B19] Haas BJ, 2013 De novo transcript sequence reconstruction from RNA-seq using the Trinity platform for reference generation and analysis. Nat Protoc. 8:1494–1512. 23845962 10.1038/nprot.2013.084 PMC3875132

[evx096-B20] Hoff KJ, Lange S, Lomsadze A, Borodovsky M, Stanke M. 2016 BRAKER1: unsupervised RNA-Seq-based genome annotation with GeneMark-ET and AUGUSTUS. Bioinformatics. 32:767–769. 26559507 10.1093/bioinformatics/btv661 PMC6078167

[evx096-B21] Huang X, Madan A. 1999 CAP3: a DNA sequence assembly program. Genome Res. 9:868–877. 10508846 10.1101/gr.9.9.868 PMC310812

[evx096-B22] Huang S, 2012 HaploMerger: reconstructing allelic relationships for polymorphic diploid genome assemblies. Genome Res. 22:1581–1588. 22555592 10.1101/gr.133652.111 PMC3409271

[evx096-B23] Jorgensen JP, 2006 Adrm1, a putative cell adhesion regulating protein, is a novel proteasome-associated factor. J Mol Biol. 360:1043–1052. 16815440 10.1016/j.jmb.2006.06.011

[evx096-B24] Kim D, 2013 TopHat2: accurate alignment of transcriptomes in the presence of insertions, deletions and gene fusions. Genome Biol. 14:R36. 23618408 10.1186/gb-2013-14-4-r36 PMC4053844

[evx096-B25] Kim JY, Kim YM, Cho SK, Choi KS, Cho M. 2008 Noble tandem-repeat galectin of Manila clam *Ruditapes philippinarum* is induced upon infection with the protozoan parasite *Perkinsus olseni*. Dev Comp Immunol. 32:1131–1141. 18440068 10.1016/j.dci.2008.03.002

[evx096-B26] Kishore U, Reid KB. 2000 C1q: structure, function, and receptors. Immunopharmacology 49:159–170. 10904115 10.1016/s0162-3109(00)80301-x

[evx096-B27] Larkin MA, 2007 Clustal W and Clustal X version 2.0. Bioinformatics 23:2947–2948. 17846036 10.1093/bioinformatics/btm404

[evx096-B28] Li B, Dewey CN. 2011 RSEM: accurate transcript quantification from RNA-Seq data with or without a reference genome. BMC Bioinformatics. 12:323. 21816040 10.1186/1471-2105-12-323 PMC3163565

[evx096-B29] Li Y, 2016 Transcriptome sequencing and comparative analysis of ovary and testis identifies potential key sex-related genes and pathways in scallop *Patinopecten yessoensis*. Mar Biotechnol. 18:1–13. 27234819 10.1007/s10126-016-9706-8

[evx096-B30] Liu HH, Xiang LX, Shao JZ. 2014a A novel C1q-domain-containing (C1qDC) protein from *Mytilus coruscus* with the transcriptional analysis against marine pathogens and heavy metals. Dev Comp Immunol. 44:70–75. 24296435 10.1016/j.dci.2013.11.009

[evx096-B31] Liu N, 2014b Effects of benzo(a)pyrene on differentially expressed genes and haemocyte parameters of the clam *Venerupis philippinarum*. Ecotoxicology 23:122–132. 24370816 10.1007/s10646-013-1157-7

[evx096-B32] Livingstone DR. 2001 Contaminant-stimulated reactive oxygen species production and oxidative damage in aquatic organisms. Mar Pollut Bull. 42:656–666. 11525283 10.1016/s0025-326x(01)00060-1

[evx096-B33] Lowe TM, Eddy SR. 1997 tRNAscan-SE: a program for improved detection of transfer RNA genes in genomic sequence. Nucleic Acids Res. 25:955–964. 9023104 10.1093/nar/25.5.955 PMC146525

[evx096-B34] Luo R, 2012 SOAPdenovo2: an empirically improved memory-efficient short-read de novo assembler. Gigascience 1:18. 23587118 10.1186/2047-217X-1-18 PMC3626529

[evx096-B35] Martin JA, Wang Z. 2011 Next-generation transcriptome assembly. Nat Rev Genet. 12:671–682. 21897427 10.1038/nrg3068

[evx096-B36] McCoy RC, 2014 Illumina TruSeq synthetic long-reads empower de novo assembly and resolve complex, highly-repetitive transposable elements. PLoS ONE. 9:e106689. 25188499 10.1371/journal.pone.0106689 PMC4154752

[evx096-B37] Milan M, 2011 Transcriptome sequencing and microarray development for the Manila clam, *Ruditapes philippinarum*: genomic tools for environmental monitoring. BMC Genomics. 12:234. 21569398 10.1186/1471-2164-12-234 PMC3107815

[evx096-B38] Mitchell A, 2015 The InterPro protein families database: the classification resource after 15 years. Nucleic Acids Res. 43:D213–D221. 25428371 10.1093/nar/gku1243 PMC4383996

[evx096-B39] Moreira R, 2012 Transcriptomics of in vitro immune-stimulated hemocytes from the Manila clam *Ruditapes philippinarum* using high-throughput sequencing. PLoS ONE. 7:e35009. 22536348 10.1371/journal.pone.0035009 PMC3334963

[evx096-B40] Nam B-H, 2016 Transcriptome analysis revealed changes of multiple genes involved in *Haliotis discus* hannai innate immunity during Vibrio parahemolyticus infection. PLoS ONE. 11:e0153474. 27088873 10.1371/journal.pone.0153474 PMC4835058

[evx096-B41] Nawrocki EP, Eddy SR. 2013 Infernal 1.1: 100-fold faster RNA homology searches. Bioinformatics 29:2933–2935. 24008419 10.1093/bioinformatics/btt509 PMC3810854

[evx096-B42] Nawrocki EP, 2015 Rfam 12.0: updates to the RNA families database. Nucleic Acids Res. 43:D130–D137. 25392425 10.1093/nar/gku1063 PMC4383904

[evx096-B43] Ondov BD, Bergman NH, Phillippy AM. 2011 Interactive metagenomic visualization in a Web browser. BMC Bioinformatics. 12:385. 21961884 10.1186/1471-2105-12-385 PMC3190407

[evx096-B44] Paillard C, Allam B, Oubella R. 2004 Effect of temperature on defense parameters in manila clam *Ruditapes philippinarum* challenged with *Vibrio tapetis*. Dis Aquat Organ. 59:249–262. 15264721 10.3354/dao059249

[evx096-B45] Park KI, Ngo TT, Choi SD, Cho M, Choi KS. 2006 Occurrence of *Perkinsus olseni* in the Venus clam *Protothaca jedoensis* in Korean waters. J Invertebr Pathol. 93:81–87. 16764886 10.1016/j.jip.2006.04.007

[evx096-B46] Park KI, 2010 Isolation and identification of *Perkinsus olseni* from feces and marine sediment using immunological and molecular techniques. J Invertebr Pathol. 105:261–269. 20691188 10.1016/j.jip.2010.07.006

[evx096-B47] Patnaik BB, 2016 Sequencing, de novo assembly, and annotation of the transcriptome of the endangered freshwater pearl bivalve, *Cristaria plicata*, provides novel insights into functional genes and marker discovery. PLoS ONE. 11:e0148622. 26872384 10.1371/journal.pone.0148622 PMC4752248

[evx096-B48] Pertea G, 2003 TIGR Gene Indices clustering tools (TGICL): a software system for fast clustering of large EST datasets. Bioinformatics 19:651–652. 12651724 10.1093/bioinformatics/btg034

[evx096-B49] Philipp EE, 2012 Massively parallel RNA sequencing identifies a complex immune gene repertoire in the lophotrochozoan *Mytilus edulis*. PLoS ONE. 7:e33091. 22448234 10.1371/journal.pone.0033091 PMC3308963

[evx096-B50] Price AL, Jones NC, Pevzner PA. 2005 De novo identification of repeat families in large genomes. Bioinformatics 21(Suppl 1):i351–i358. 15961478 10.1093/bioinformatics/bti1018

[evx096-B51] Pruitt KD, Tatusova T, Maglott DR. 2007 NCBI reference sequences (RefSeq): a curated non-redundant sequence database of genomes, transcripts and proteins. Nucleic Acids Res. 35:D61–D65. 17130148 10.1093/nar/gkl842 PMC1716718

[evx096-B52] Quevillon E, 2005 InterProScan: protein domains identifier. Nucleic Acids Res. 33:W116–W120. 15980438 10.1093/nar/gki442 PMC1160203

[evx096-B53] Rao R, 2015 RNA-seq analysis of *Macrobrachium rosenbergii* hepatopancreas in response to *Vibrio parahaemolyticus* infection. Gut Pathog. 7:1. 25922623 10.1186/s13099-015-0052-6 PMC4411767

[evx096-B54] Schick JM, Gnaiger E, Widdows J, Bayne BL, de Zwaan A. 1986 Activity and metabolism in the mussel *Mytilus edulis* L.during intertidal hypoxia and aerobic recovery. Physiol Zool. 59:627–642.

[evx096-B55] Simakov O, 2013 Insights into bilaterian evolution from three spiralian genomes. Nature 493:526–531. 23254933 10.1038/nature11696 PMC4085046

[evx096-B56] Slater GS, Birney E. 2005 Automated generation of heuristics for biological sequence comparison. BMC Bioinformatics. 6:31. 15713233 10.1186/1471-2105-6-31 PMC553969

[evx096-B57] Small KS, Brudno M, Hill MM, Sidow A. 2007 A haplome alignment and reference sequence of the highly polymorphic *Ciona savignyi* genome. Genome Biol. 8:R41. 17374142 10.1186/gb-2007-8-3-r41 PMC1868934

[evx096-B58] Smit AHR, Green P. 1996–2010. RepeatMasker Open-4.0.5.

[evx096-B59] Stanke M, Steinkamp R, Waack S, Morgenstern B. 2004 AUGUSTUS: a web server for gene finding in eukaryotes. Nucleic Acids Res. 32:W309–W312. 15215400 10.1093/nar/gkh379 PMC441517

[evx096-B60] Takeuchi T, 2012 Draft genome of the pearl oyster Pinctada fucata: a platform for understanding bivalve biology. DNA Research. 19:117–130. 22315334 10.1093/dnares/dss005 PMC3325083

[evx096-B61] Takeuchi T, 2016 Bivalve-specific gene expansion in the pearl oyster genome: implications of adaptation to a sessile lifestyle. Zool Lett. 2:1. 10.1186/s40851-016-0039-2 PMC4759782 26900483

[evx096-B62] Teaniniuraitemoana V, 2014 Gonad transcriptome analysis of pearl oyster *Pinctada margaritifera*: identification of potential sex differentiation and sex determining genes. BMC Genomics. 15:1. 24942841 10.1186/1471-2164-15-491 PMC4082630

[evx096-B63] Tom Tang Y, 2005 The complete complement of C1q-domain-containing proteins in Homo sapiens. Genomics 86:100–111. 15953544 10.1016/j.ygeno.2005.03.001

[evx096-B64] UniProt C. 2015 UniProt: a hub for protein information. Nucleic Acids Res. 43:D204–D212. 25348405 10.1093/nar/gku989 PMC4384041

[evx096-B65] Untergasser A, 2012 Primer3 – new capabilities and interfaces. Nucleic Acids Res. 40:e115. 22730293 10.1093/nar/gks596 PMC3424584

[evx096-B66] Voskoboynik A, 2013 The genome sequence of the colonial chordate, *Botryllus schlosseri*. Elife 2:e00569. 23840927 10.7554/eLife.00569 PMC3699833

[evx096-B67] Wang L, 2012 A C1q domain containing protein from scallop *Chlamys farreri* serving as pattern recognition receptor with heat-aggregated IgG binding activity. PLoS ONE. 7:e43289. 22905248 10.1371/journal.pone.0043289 PMC3419688

[evx096-B68] Waterhouse AM, Procter JB, Martin DM, Clamp M, Barton GJ. 2009 Jalview Version 2 – a multiple sequence alignment editor and analysis workbench. Bioinformatics 25:1189–1191. 19151095 10.1093/bioinformatics/btp033 PMC2672624

[evx096-B69] Yue X, Nie Q, Xiao G, Liu B. 2015 Transcriptome analysis of shell color-related genes in the clam *Meretrix meretrix*. Mar Biotechnol. 17:364–374. 25680512 10.1007/s10126-015-9625-0

[evx096-B70] Zhang G, 2012 The oyster genome reveals stress adaptation and complexity of shell formation. Nature 490:49–54. 22992520 10.1038/nature11413

