# Supplementary material for: The Whole-Genome and Transcriptome of the Manila Clam (Ruditapes philippinarum)
Source: Genome Biol Evol. 2017 May 13;9(6):1487–98. doi: 10.1093/gbe/evx096 (PMC5499747; doi:10.1093/gbe/evx096)
Supplement: Supplementary Data [file evx096_Supp.zip › Supplementary Figures.pdf]

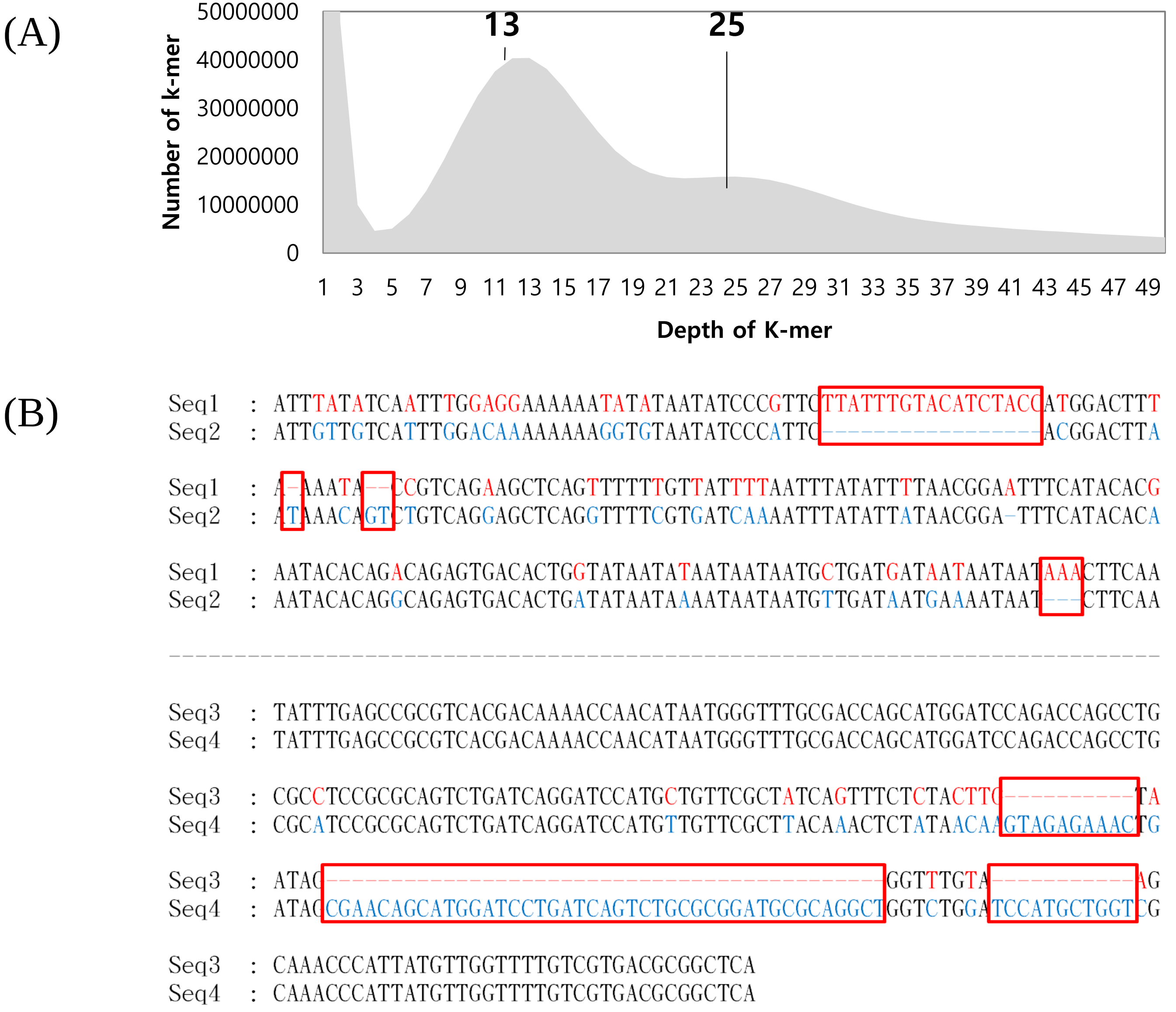

Figure S1. Estimation of the *R. philippinarum* genome size based on 17-mer analysis.
(A) The *x*-axis and the *y*-axis denotes the depth (X) and the proportion, respectively, i.e., the frequency at that depth divided by the total frequency at all depths. (B) Example of Heterozygous features in the *R. philippinarum* genome. By performing sequence comparison using large-scale genome alignment tool (LASTZ), high-rate of hererozygosity between allele were defined. Red and blue letters indicate sequence difference and red boxes indicate micro-insertion or deletions between two allele.

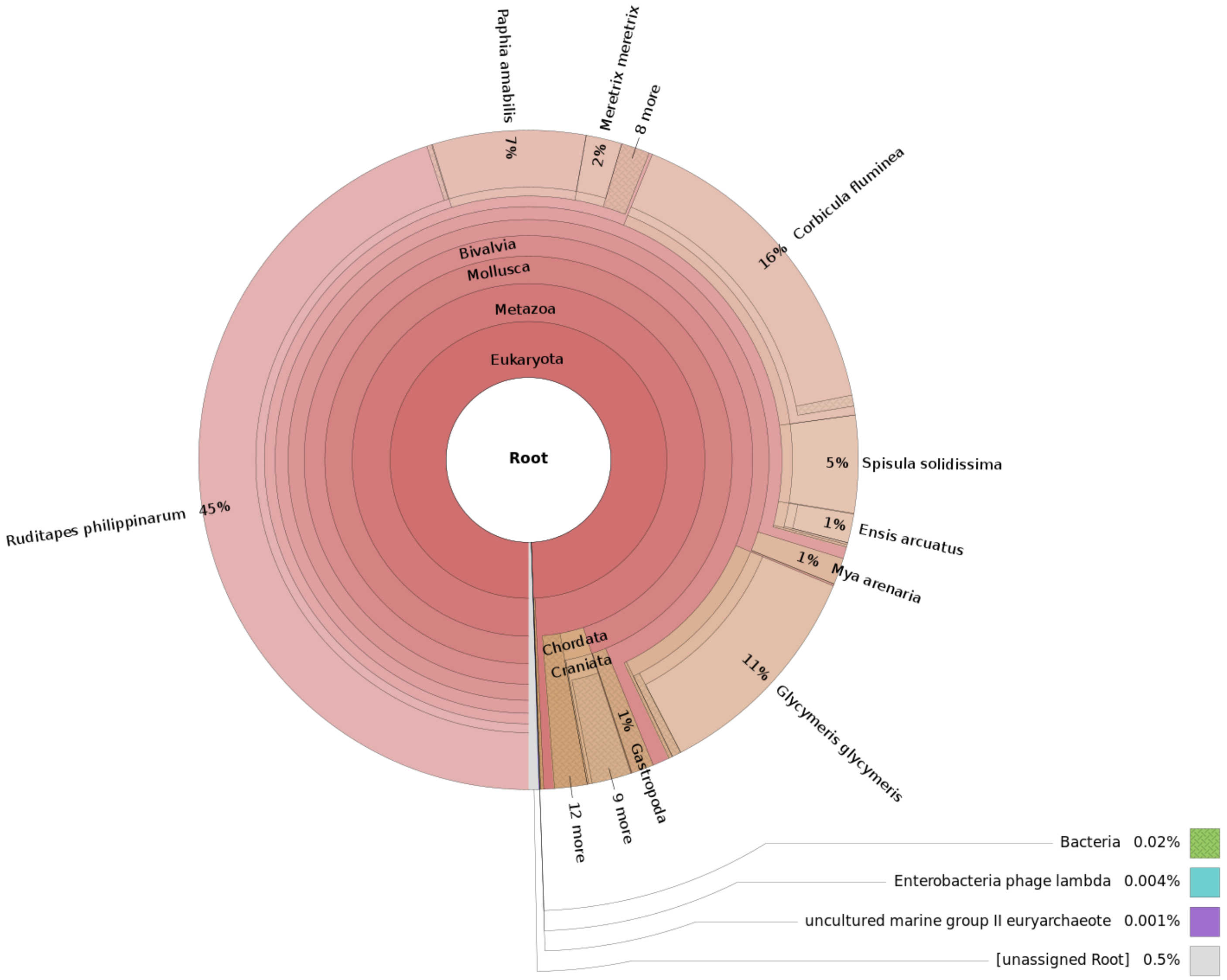

Figure S2. Krona chart for taxonomy profiling of *R. philippinarum*.
All aligned contigs and scaffolds to NCBI non-redundant nucleotide database (nt database) were assigned to Krona. The abundance of the taxonomic groups corresponds to the percentage of spectra based on sequence identity.

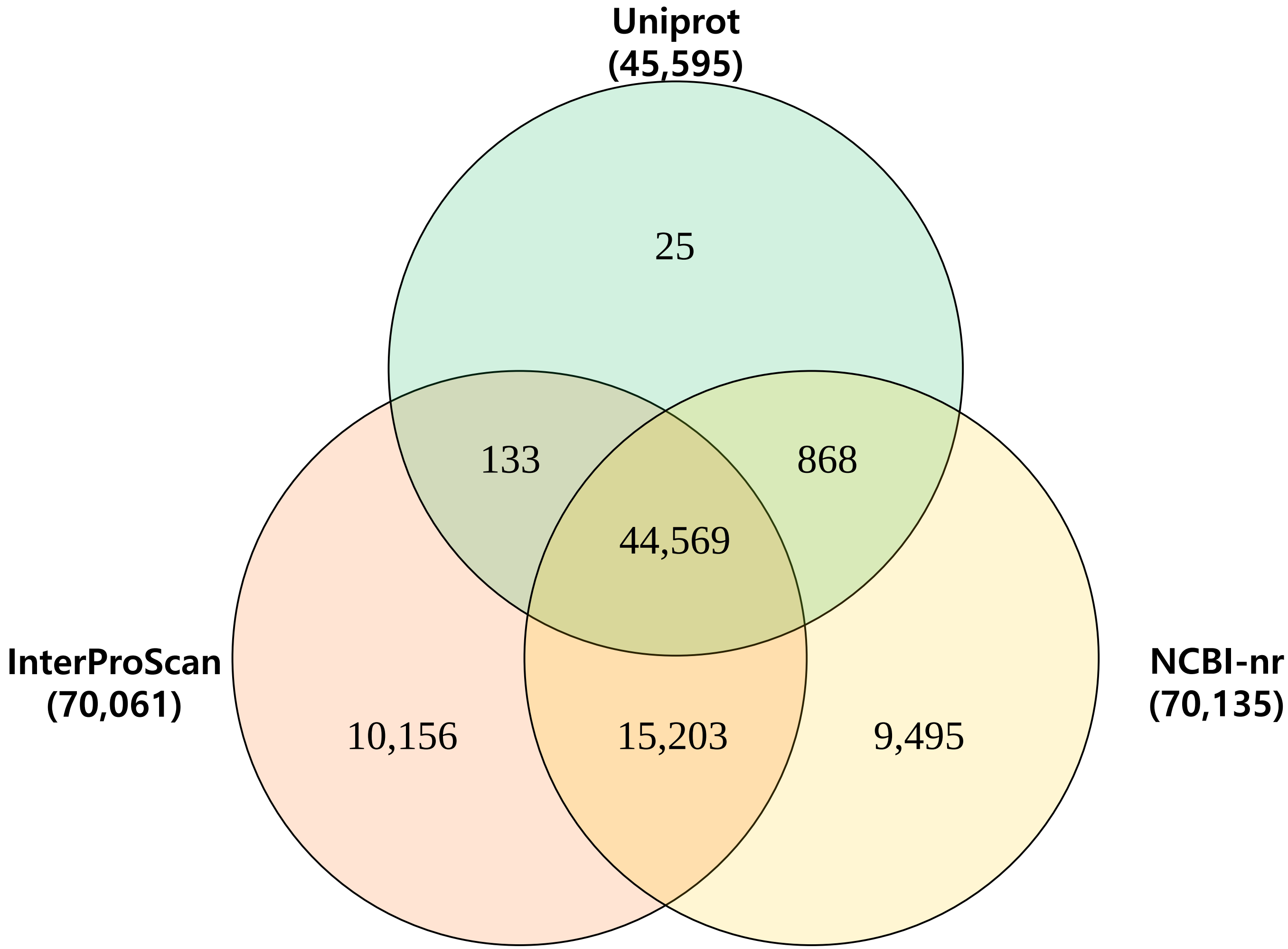

Figure S3. Summary of *R. philippinarum* gene prediction with three identifier (Uniprot, InterProScan, and NCBI-nr databse).
The number of unique and shared gene models from three databases represent in the Venn diagram.

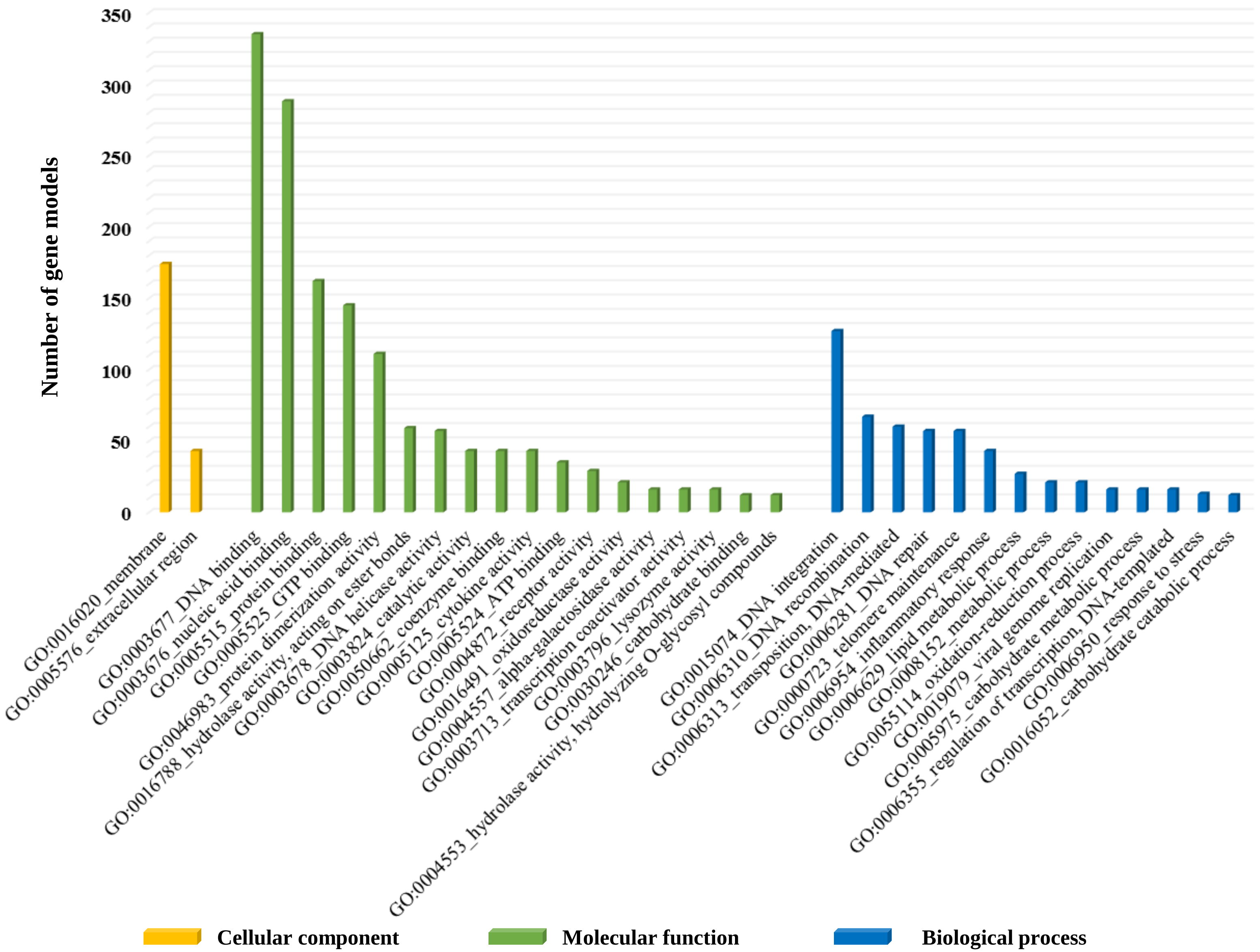

Figure S4. Gene Ontology (GO) categories of 22 gene families, highly enriched in *R. philippinarum*.
The results is summarized in three main categories: cellular components (yellow), molecular function (green), and biological process (blue). The X axis denoted the related functional categories and the Y axis denoted the number of gene models, which are associated with each GO categories.
