# Supplementary figures and images for: The Whole-Genome and Transcriptome of the Manila Clam (Ruditapes philippinarum)
Source: Genome Biol Evol. 2017 May 13;9(6):1487–98. doi: 10.1093/gbe/evx096 (PMC5499747; doi:10.1093/gbe/evx096)

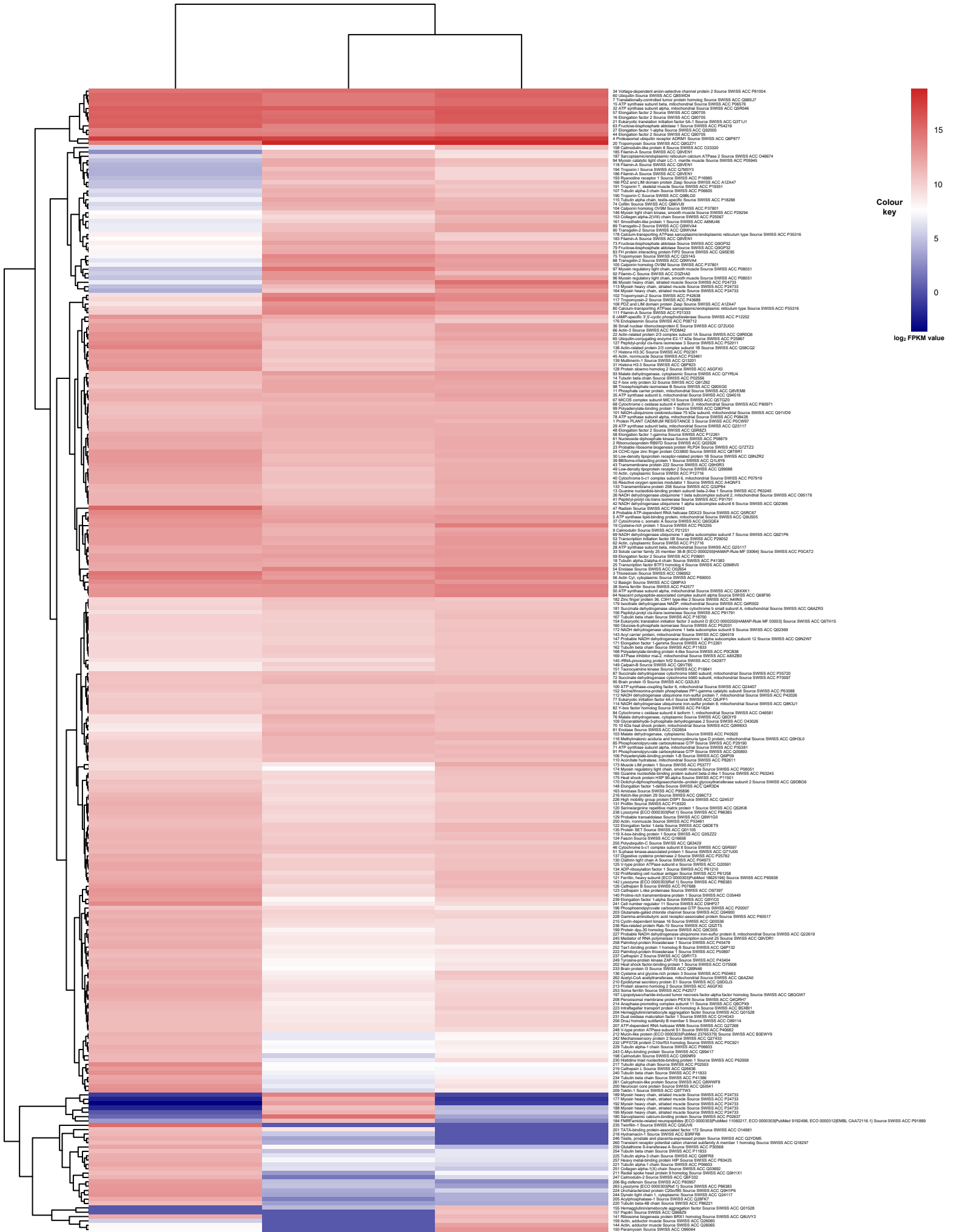

Gill:log2

Adductor:muscle:log2

Foot:log2

Colour

log<sub>2</sub> FPKM value

Supplement: Supplementary Data [file evx096_Supp.zip › Supplementary Figure S5.pdf]
